# Positive Association Between Online Attention and the Bibliometric Impact of Shoulder Instability Publications

**DOI:** 10.1016/j.asmr.2022.04.023

**Published:** 2022-06-11

**Authors:** Youssef Abdullah, Abraham J. Mathew, Aaron Alokozai, Michaela A. Stamm, Mary K. Mulcahey

**Affiliations:** Department of Orthopaedic Surgery, Tulane University School of Medicine, New Orleans, Louisiana, U.S.A.

## Abstract

**Purpose:**

To obtain a quantifiable measure of the frequency with which a shoulder instability article is discussed online and the association with its corresponding bibliometric impact, based on the Scopus Cite Score (SCS) or Web of Science Impact Factor (WSIF).

**Methods:**

The top 100 most-mentioned articles on shoulder instability based on Altmetric Attention Score (AAS) were extracted from the Altmetric Database. Mentions within blogs, news articles and outlets, public policy, and social media platforms, such as Twitter and Facebook, were included. Study impact was assessed using SCS or WSIF. The degree of association between AAS and impact was determined using Spearman correlation, logarithmic regression, and multivariate regression.

**Results:**

The most common study designs were “Clinical Trial,” with 52 articles (49.5%), “Systematic Review” with 16 articles (15.2%), and “Review” with 10 articles (9.5%). Twitter provided more online mentions than other platforms, with the average article being discussed 27.7 times (range 0-220 times). A significant positive effect (estimate = 2.616, *P* = .0075) was observed between the AAS and WSIF, based on the logarithmic regression. Multivariate regression revealed that blogs help raise both WSIF and SCS (estimate = 7.272, *P* < .05).

**Conclusions:**

Social media and other online platforms are a strong way to disseminate information to patients. A positive association was observed between overall online attention and the bibliometric impact of an article related to shoulder instability. Clinical trials related to shoulder instability that receive online mentions, especially discussion in blogs, are more likely to be cited in the future than their counterparts.

**Clinical Relevance:**

The results of our study can guide authors as they aim to disseminate their articles. Twitter may be used as a tool to reach patients who may not venture into academic journals with current peer-reviewed articles. Further, blogs may be used to reach academic audiences and raise bibliometric impact broadly.

Online platforms, including social media and blogs, are among the most effective methods for disseminating health care information.^1^^,^^2^ Advances in internet access have begun to rapidly transform fundamental health-related communications.^3^ In orthopaedic surgery, social media use continues to increase.^4^, ^5^, ^6^ Rapidly transmitting health care information, while keeping it accessible via improved user interaction, helps patients more efficiently gain the knowledge necessary to take initiative in contributing to their treatment plans.^7^^,^^8^ In addition, the benefits of patient participation in treatment do not end at a relational level. Sepucha et al.^9^ demonstrated that patients who receive their preferred, personalized treatment have greater satisfaction and improved health care outcomes. Based on the role and benefits of online media in patient care, it is important to take into account the social media impact and online presence of articles, as those with the greatest scores are most likely to be identified and reviewed by patients.

The established standard for evaluating an article’s impact is bibliometric analysis, which quantifies citations an article receives since the date of publication.^10^ There are several measures that can be used to determine the impact of an article based on citations, including the Scopus Cite Score (SCS), used by the Scopus database, and Web of Science Impact Factor (WSIF), used by the Web of Science database.^11^^,^^12^ The SCS and the WSIF are the average number of citations per article that a journal receives over 3 years.^11^^,^^12^ They are not exact proxies for an article’s impact and are more representative of the impact of a journal’s average article. Because the SCS is calculated by Scopus and the WSIF is calculated by Web of Science, including both allows citations not included in one database, and therefore not factored into one of the scores, to be accounted for in the other.

Altmetric Attention Score (AAS) takes into account the number of times other articles cite the article in question and is an effective combined measure of an article’s online attention and impact. However, there may not always be a direct correlation between number of times cited, which is the basis of the SCS and WSIF, and AAS. For example, Celik et al. compared academic attention via citations of general oncology articles to their online attention and found no correlation between number of times cited and AAS.^6^ Even so, weak correlations between AAS and number of citations have been observed in the fields of urology, endodontology, radiology, and spine surgery, strengthening the possibility of an association between the two.^13^, ^14^, ^15^

The online availability of medical articles can influence patient understanding, including raising patient comprehension of the etiology of their disease process, rehabilitation and management, and prognosis after an intervention.^16^, ^17^, ^18^, ^19^ However, the association between online mentions of medical articles and their overall impact remains unclear in orthopaedic surgery. The purpose of this study was to obtain a quantifiable measure of the frequency with which a shoulder instability article is discussed online and the association with its corresponding bibliometric impact, based on the SCS or WSIF. We hypothesized that overall online attention likely would be associated with bibliometric impact of an article related to shoulder instability.

## Methods

### Search Strategy

The top 100 articles by AAS related to shoulder instability from October 2011 to January 2022 were identified by searching the Altmetric database for the term “shoulder instability” on January 2022.

### Data Collection

The AAS is a weighted measure based on the number and type of online mentions an article has received. Sources of online attention tracked by the Altmetric database include peer-reviewed journal articles, clinical trials, dissertations, reports, conference proceedings, online data sets, manually updated online news outlets, public policy documents, and books.^20^ Online mentions tracked by the Altmetric database are incorporated into the AAS, which adjusts for factors that could artificially inflate an article’s calculated worth (duplicate tweets and multiple mentions).^21^

In addition to the AAS, the total number of online mentions in the news, online blogs, public policy, and social media were collected from the Altmetric database for each article. Table 1 lists the social media outlets that were included along with their Altmetric Attention Score. The SCS and the WSIF were collected from Scopus and Web of Science, respectively. To effectively use these scores, each article included was assumed to perform at or near its native journal’s average. Other collected data included publication date, journal, article type, and topic for each article.

**Table 1:** Altmetric Attention Score Weights^21^

Attention Type	Points
News	8
Blog	5
Policy	3
Patent	3
Wikipedia	3
Twitter (tweets and retweets)	1
Peer review (Publons, PubPeer)	1
Weibo (not trackable since 2015, but historical data kept)	1
Google+ (not trackable since 2019, but historical data kept)	1
F1000	1
Syllabi (Open Syllabus)	1
LinkedIn (not trackable since 2014, but historical data kept)	0.5
Facebook (only a curated list of public Pages)	0.25
Reddit	0.25
Pinterest (not trackable since 2013, but historical data kept)	0.25
Q&A (Stack Overflow)	0.25
YouTube	0.25

### Statistical Analysis

R, an open-source statistical programming language and data analysis tool, was used to perform the quantitative aspects of the study. Spearman correlation, logarithmic regression, and multivariate regression were used to determine the degree of association between each type of online mention and an article’s SCS and WSIF. In addition, the AAS was also compared with SCS and WSIF using the tests described previously. Correlation strength was determined using previously established categories: weak correlations fall between 0.1 and 0.3, moderate correlations fall between 0.3 and 0.5, and strong correlations are greater than 0.5.^22^ The statistical significance cut-off was set at *P* < .05.

## Results

### Search Results

During the initial search, 15 articles were tied for the 92nd position within the top 100 articles related to shoulder instability. As a result, this study included 107 articles; however, one article could not be located during the review process, leaving 106 articles for analysis.

### Study Designs

**Fig 1:**
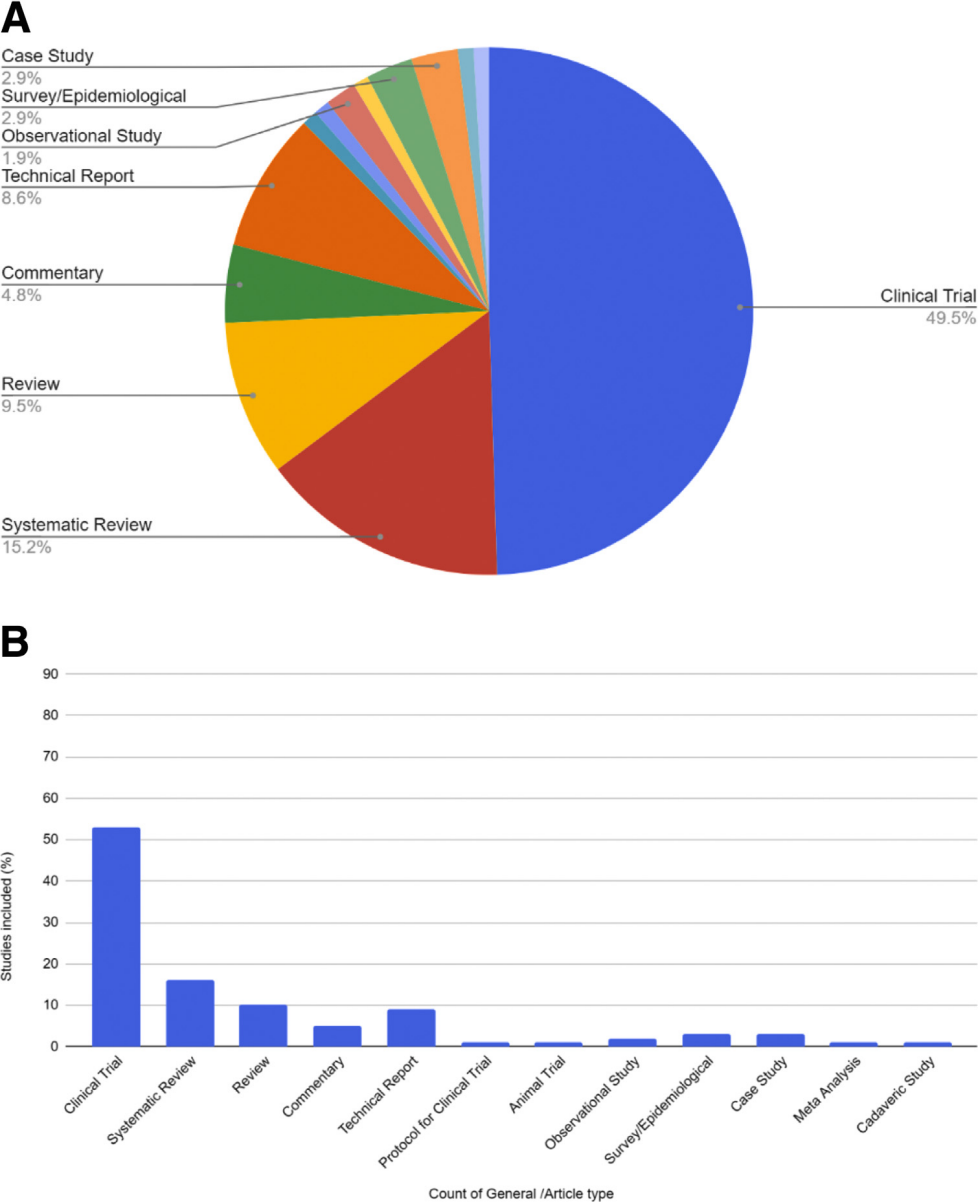
(A-B) Study design for the top 100 articles related to shoulder instability.

Among the top 100 articles related to shoulder instability, clinical trials, not including observational studies, were the most common study design (49.5%, N = 53), followed by systematic reviews (15.2%, N = 16), and review articles (9.5%, N = 10) (Fig 1 A and B).

### Online Mentions

The average AAS was 21 (range 10-104). Twitter appears to be the major contributor to an article’s AAS within the top 100 articles on shoulder instability (Table 1). Other online media platforms mentioned shoulder instability articles much less frequently compared with Twitter and Facebook (Table 2).

**Table 2:** Average Number of Online Mentions of a Top 100 Article Related to Shoulder Instability

Facebook	Twitter	News	Blogs	Policy
1.8 (range 0-24)	27.7 (range 0-220)	0.4 (range 0-1)	0.0 (range 0-1)	0.0 (range 0-1)

To assess the association between an article’s online mentions and the impact of the average article within the journal in which it was published, Spearman correlation, logarithmic regression, and multivariate regression were performed.

### Spearman Correlation

The correlation coefficients between online mentions and impact of an article within the journal in which it was published are included in Table 3.

**Table 3:** Correlation Coefficients Between Media Outlets and Impact Measures

	WSIF	SCS
Facebook	0.1172	0.1366
Twitter	0.2229∗	–0.0934
News	0.0186	0.1038
Blogs	0.4912∗	0.4043∗
Policy documents	0.0193	0.0754
Altmetric Score	0.3320∗	0.0598

SCS, Scopus Cite Score; WSIF, Web of Science Impact Factor.

∗ Weakly correlated.

### Logarithmic Regression

A logarithmic transformation was applied to each of the included media outlets and the AAS. Linear regression was performed using the log-transformed variables and the impact measured by both the SCS and the WSIF (Table 4). The only significant effect was observed between the AAS and WSIF. Based on the results of the logarithmic regression, for every 1% increase in AAS, there is a 0.0262-point increase in WSIF (*P* = .0075). In addition, the model generated via logarithmic regression only explains about 8.1% of the variance seen in WSIF (R^2^ = 0.0811).

**Table 4:** Multivariate Regression: Web of Science Impact Factor

	Estimate	Standard Error	t Value	*P* Value
Twitter	0.032	0.021	1.576	.119
Facebook	–0.098	0.146	–0.674	.502
News	0.329	0.594	0.554	.581
Blogs	9.911	2.083	4.758	8.40 × 10^-6^∗
Policy	1.917	4.340	0.442	.66

∗ Significant effect (*P* < .05).

### Multivariate Regression

Multivariate regression was performed to evaluate the effect of each online media outlet on bibliometric impact (WSIF and SCS), while accounting for the effects of the other online media outlets. The multivariate regression model demonstrated that online mentions in blogs were the only type of online mention that significantly affected both WSIF and SCS (Tables 4 and 5).

**Table 5:** Multivariate Regression—Scopus Cite Score

	Estimate	Standard Error	t Value	*P* Value
Twitter	–0.018	0.0129	–1.527	.13
Facebook	0.105	0.115	0.917	.362
News	0.289	0.468	0.617	.538
Blogs	7.272	1.738	4.183	6.46 × 10^-5^∗
Policy	2.808	3.685	0.762	.448

∗ Significant effect (*P* < .05).

Being mentioned in blogs appears to lead to a 9.911-point increase in the WSIF. While this effect size is large, only 5 of the articles included here (4.7%) were mentioned in blogs. In addition, the multivariate regression model only explains 26.6% of the variance seen in the WSIF (R^2^ = 0.2657).

Table 5 lists the estimated effects of each type of online mention on SCS and their statistical significance based on the multivariate regression model. According to the model, one online blog mention leads to a 7.272-point increase in the SCS. However, this model only explains about 20.1% of the variance seen in the SCS (R^2^ = 0.2011). Similar to the results relating to WSIF, the percentage of variance explained by the model also implies that factors other than online mentions of articles influence a journal's impact based on the SCS. Therefore, like the WSIF, it is unclear whether blogs alone impact SCS despite the statistically significant result.

## Discussion

Clinical trials made up the majority of the top 100 articles related to shoulder instability. An association was revealed between AAS and both bibliometric impact measures (WSIF and SCS). Blogs appear to have a significant positive effect on both bibliometric impact measures.

Clinical trials were the most common study design within the top 100 shoulder instability articles, making up 49.5% of the total. This indicates that clinical trials receive more online attention than other article types. The observed increased attention received by clinical trials is consistent with other publications in orthopaedics. For instance, Parrish et al.^15^ determined that clinical trials were the most common article type within the top 100 articles by AAS in spinal surgery. The prominence of clinical trials observed here for articles related to shoulder instability is also consistent with the results observed in a study by Barbic et al.,^23^ in which the authors analyzed the Altmetric performance of the 200 most-cited emergency medicine articles in the top 10 emergency medicine journals according to the 2011 Journal citation report. However, there are some specialties with differing results. For example, an endodontologic Altmetric study by Kolahi et al.^13^ determined that meta-analyses and systematic reviews received the most online attention. The origin of the discrepancies within article type popularity between specialties is currently unknown and holds potential for future research.

The most common online mention outlet within the top 100 was Twitter. Parrish et al.^15^ also determined that Twitter is the most common outlet within the top 100 spinal surgery articles by online attention. The prevalence of Twitter in disseminating research articles is not limited to articles within orthopaedic surgery. A recent study by Wang et al. evaluated the top 100 neurosurgery articles and found that Twitter was the most prominent source of online attention.^3^ Overall, Twitter appears to be a ubiquitous source of online media attention for medical articles. Future research may investigate patient preferences of how to access online information and their understanding of the articles shared.

A weak correlation was observed between AAS and both measures of bibliometric impact used in this study. Similarly, weak correlations between AAS and the bibliometric impact measures were observed in Altmetric papers within urology, endodontology, radiology, and spine surgery.^13^, ^14^, ^15^^,^^24^ Because the discovered association is weak, it was difficult to determine definitively if there was an association between AAS and bibliometric impact. Therefore, this association was further explored via logarithmic regression. When considering the aforementioned studies’ results, it is important to note that these studies did not assess the correlation between AAS, WSIF, and SCS scores. Instead, citation count was the primary focus. Although citation count is a different metric, WSIF and SCS are calculated based on citation count, making these results analogous, although not ideal one-to-one comparisons.

To definitively assess the weak correlation between the log-transformed variables, AAS scores, and bibliometric impact using WSIF and SCS, a linear regression was performed. The results disclosed a significant effect between AAS and WSIF (for every 10% increase in AAS, there is a 0.262-point increase in WSIF). The study by Parrish et al.^15^ evaluating the top 100 spinal surgery articles by AAS and the social media outlets used for their dissemination did not observe a similar statistically significant relationship between AAS and WSIF, making the relationship between AAS and WSIF potentially unique to shoulder instability. Furthermore, the same effect has not been observed in the previously discussed studies focused on the AAS of articles in other medical fields (e.g., urology, endodontology, and radiology).^13^, ^14^, ^15^^,^^24^

The models generated by the multivariate regression revealed that blogs were the only type of online mention that had a significant effect on both WSIF and SCS. However, blogs were relatively rare when compared with the other online media types. Therefore, it is possible that these results occurred due to other factors. In addition, there is evidence in the literature that supports the positive effects of blogs on the citation of articles. For instance, a multidisciplinary study of scientific articles conducted by Costas et al.^25^ on the relationship between Altmetrics and citations determined that blogs play a crucial role in identifying highly cited publications. More specifically, the study determined that online attention in blogs had a stronger correlation with citations than in other media outlets.

### Limitations

There are several limitations to this study. First, there is a temporal association between online attention and the date of a manuscript’s publication. More recent articles will receive more attention. Second, the AAS is susceptible to change, and an article’s attention can decline over time.^17^ However, older articles are more likely to be cited than recent publications and will carry more impact than recent articles. Therefore, the long-term bibliometric impact of recently published articles is unclear and is susceptible to change. Some online media outlets, such as Instagram, were not included in the calculation of AAS by Altmetric.^20^ As a result, AAS is not a perfectly comprehensive measure of online attention. Finally, factors beyond online attention may play a role in defining an article’s bibliometric impact. For instance, the notoriety and the nationality of the first author can play a role in how often an article is cited.^26^ Therefore, although the findings offered by this study propose possible advantages of specific online outlets, such as blogs and Twitter, in promoting bibliometric impact, their effectiveness may be mediated by other factors.

## Conclusions

Social media and other online platforms are a strong way to disseminate information to patients. A positive association was observed between overall online attention and the bibliometric impact of an article related to shoulder instability. Clinical trials related to shoulder instability that receive online mentions, especially discussion in blogs, are more likely to be cited in the future than their counterparts.

## Supplementary Material

ICMJE author disclosure forms
